# Hybrid dose calculation: a dose calculation algorithm for microbeam radiation therapy

**DOI:** 10.1088/1361-6560/aaa705

**Published:** 2018-02-13

**Authors:** Mattia Donzelli, Elke Bräuer-Krisch, Uwe Oelfke, Jan J Wilkens, Stefan Bartzsch

**Affiliations:** 1 The European Synchrotron Radiation Facility, 71 Avenue des Martyrs 38000, Grenoble, France; 2 The Institute of Cancer Research, 15 Cotswold Road, Sutton, London SM2 5NG, United Kingdom; 3 Department of Radiation Oncology, Klinikum rechts der Isar, Technical University of Munich, Ismaninger Straße 22, 81675 Munich, Germany; 4 Author to whom any correspondence should be addressed.; mail@donzelli.eu

**Keywords:** microbeam radiation therapy, dose calculation, Monte Carlo, dose kernel convolution

## Abstract

Microbeam radiation therapy (MRT) is still a preclinical approach in radiation oncology that uses planar micrometre wide beamlets with extremely high peak doses, separated by a few hundred micrometre wide low dose regions. Abundant preclinical evidence demonstrates that MRT spares normal tissue more effectively than conventional radiation therapy, at equivalent tumour control. In order to launch first clinical trials, accurate and efficient dose calculation methods are an inevitable prerequisite.

In this work a hybrid dose calculation approach is presented that is based on a combination of Monte Carlo and kernel based dose calculation. In various examples the performance of the algorithm is compared to purely Monte Carlo and purely kernel based dose calculations.

The accuracy of the developed algorithm is comparable to conventional pure Monte Carlo calculations. In particular for inhomogeneous materials the hybrid dose calculation algorithm out-performs purely convolution based dose calculation approaches. It is demonstrated that the hybrid algorithm can efficiently calculate even complicated pencil beam and cross firing beam geometries. The required calculation times are substantially lower than for pure Monte Carlo calculations.

## 1. Introduction

Microbeam radiation therapy (MRT) is an innovative approach in radiation therapy that utilizes arrays of a few tens of micrometre wide and a few 100 *μ*m spaced planar beamlets with extremely high doses of several hundred Grays in the radiation peaks and doses below the tissue tolerance level between the beamlets in the microbeam valleys. A modulation of radiation doses on the micrometre scale, also referred to as spatial fractionation, has proven to significantly reduce side effects in normal tissue (Slatkin *et al* 1992, Laissue *et al* 2001, Serduc *et al* 2008, Bouchet *et al* 2010), even at high peak doses, as compared to conventional radiation therapy at equal tumour control (Laissue *et al* 1998, Regnard *et al* 2008, Bouchet *et al* 2010).

In order to deliver dose profiles with sharp beam penumbras in a clinical MRT treatment, radiation with low or short ranged scattering, low beam divergence and high dose rates is required. Furthermore, the dose fall-off with depth in water should be sufficiently flat, to allow treatment of deep-seated tumours. Photon beams with a kinetic energy of approximately 100 keV exhibit promising properties for the generation of microbeams. Currently, however, only large third generation synchrotrons such as the European Synchrotron (ESRF) in Grenoble, France provide acceptable beam parameters for a clinical application of MRT (Fournier *et al* 2016).

At the biomedical beamline ID 17 of the ESRF a multislit collimator located at 41.7 m distance from the wiggler source shapes 50 *µ*m wide and 400 *µ*m centre-to-centre spaced microbeams (Bräuer-Krisch *et al* 2009). Various absorption filters modify the spectrum of the synchrotron beam such that the the final treatment beam has its maximum intensity at 83 keV and a mean energy of around 100 keV (Siegbahn *et al* 2006, Crosbie *et al* 2015).

Absorption and scattering of photons in an energy regime of around 100 keV is dominated by Compton scattering, photoelectric absorption and Rayleigh scattering. The mean free path length of photons in water, i.e. the mean path a photon travels without interaction, is of the order of centimetres. At the site of a photon interaction secondary electrons are generated. Secondary electrons rapidly lose their energy in collisions, where Coulomb scattering is the dominant process. Contributions of radiative energy loss are extremely low. The range of electrons is up to a few hundred micrometres (Berger *et al* 2010).

Until now dose distributions in MRT were mainly calculated in Monte Carlo simulations. Various Monte Carlo codes have been used for this purpose, among others Geant4 (Stepanek *et al* 2000, Cornelius *et al* 2014), EGS4 (Orion *et al* 2000, De Felici *et al* 2007) and PENELOPE (Siegbahn *et al* 2006, Martínez-Rovira *et al* 2010, Martínez-Rovira *et al* 2012, Prezado *et al* 2012). Comparisons of different Monte Carlo codes in MRT were performed by De Felici *et al* (2008) and Spiga *et al* (2007). Most of these simulations used simplified patient geometries and models of the radiation source. A challenge for Monte Carlo simulation of MRT dose distributions are the required resolution on a micrometre scale and the large dose differences between microbeam peak and valley doses. To keep the statistical uncertainties of computed doses low, a large number of particle histories, and consequently long calculation times, are required. Particularly for cross firing beam geometries (Miura *et al* 2006, Bouchet *et al* 2010, Serduc *et al* 2010) or pencil beams (Fernandez-Palomo *et al* 2013, Schültke *et al* 2013), where high spatial resolution is required in all dimensions Monte Carlo simulations become extremely time consuming. Cornelius *et al* (2014) recently estimated a total calculation time of 10 h on 100 CPU cores in parallel to gain sufficient statistics.

An alternative approach to Monte Carlo simulations are convolution based dose calculation algorithms. These algorithms usually separate the energy transport of the primary unscattered beam from energy transport by scattering photons and electrons. Scatter kernels are derived, which describe the mean spatial distribution of absorbed energy from secondary particles created in primary photon interaction at the origin (Ahnesjö *et al* 1987). These kernels are convoluted over the primary interaction frequency per volume element *d*^3^*r* of the primary photon beam. For photon microbeams of around 100 keV kinetic energy, electron and photon scatter kernels can be separated (Bartzsch and Oelfke 2013). Kernel based dose calculation in MRT is substantially faster than Monte Carlo simulations and dose calculations for a typical MRT field of 2 cm side length in an anthropomorphic target can be accomplished within less than 5 min on a conventional PC (3.4 GHz processor, 8 GByte RAM) (Debus *et al* 2017).

The drawbacks of kernel based dose calculation methods are inaccuracies in the calculation of scattered photon transport close to material boundaries. Scatter kernels are computed for homogeneous material and changes in the scattering close to material interfaces are ignored. Therefore, convolution based dose calculation can lead to inaccurate dose estimates close to material interfaces. For MRT this is particularly problematic in valley regions, where mainly scatter photons contribute to the absorbed energy (Debus *et al* 2017). Moreover, low energy photon beams face more drastic variations in radiological parameters such as absorption coefficients in different anatomic structures when compared to MeV photon beams used in conventional radiation therapy.

Here we present a new hybrid method that combines the advantage of Monte Carlo based dose calculation to accurately calculate photon scattering and the advantage of convolution based dose calculation to efficiently calculate electron energy absorption on a micrometre scale. The newly developed technique is more precise than a pure dose kernel convolution approach in the transport of scattered photons, while being substantially faster than current Monte Carlo algorithms.

Accurate dose calculations additionally rely on an adequate model of the radiation source and patient information in the form of medical imaging, usually CT images. Detailed simulation of the ID17 medical beam line (Martínez-Rovira *et al* 2012) and investigations on properties of synchrotron radiation (De Felici *et al* 2005, Hugtenburg *et al* 2010) were performed in the past and are integrated in the presented algorithms.

## 2. Methods

### 2.1. General concept of a hybrid algorithm

In MRT, the microbeam peak dose originates from the absorption of electrons produced in interactions of the primary unscattered photon beam, while the valley dose comprises the energy absorbed from scattered photons and electrons scattering from the peak into the valley.

**Figure 1.**
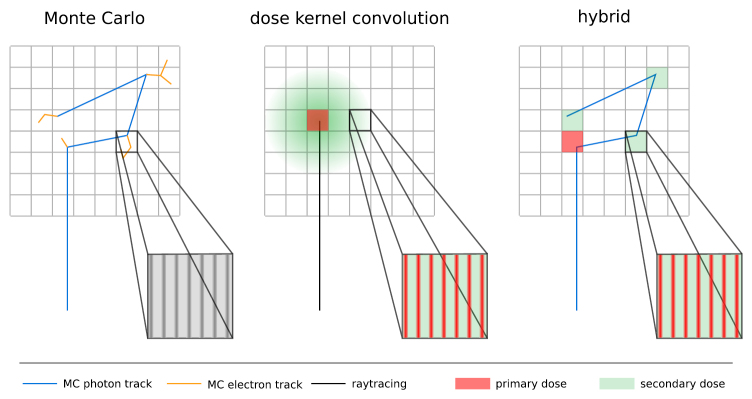
The hybrid dose calculation algorithm for MRT inherits photon transport from Monte Carlo dose calculation and electron transport from dose kernel convolution based dose calculation. The energy transfer from photons to electrons is separated into primary (red) and secondary (green) scattering. The primary dose is further processed with dose kernel convolution for electron transport (see figure 2).

The hybrid algorithm separates photon and electron mediated energy transport. The photon transport is calculated with Monte Carlo simulation. These simulations are performed on a millimetre scale based on the voxel size of an underlying CT image and only take photon interactions into account. In each voxel the transfer of energy from primary and scattered photons to secondary electrons is scored independently, resulting in a primary and scattered photon dose cube. This procedure is illustrated in figure 1 alongside the Monte Carlo and pure convolution algorithm.

The electron energy transport is calculated in a convolution based dose calculation algorithm. The range of electrons is usually smaller than the size of a typical CT voxel. Within a voxel the material is assumed to be homogeneous. Since information on tissue inhomogeneities originate from the CT image, information on smaller structures are unknown anyway.

**Figure 2.**
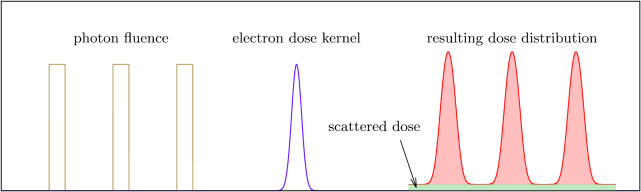
The peak dose distribution is calculated by convolving the photon fluence with analytic electron dose kernels. The integrated dose under the microbeam peaks (shaded in red) must be equal to the dose deposited in primary photon interactions. The dose from scattered photon interactions (green) is homogeneously distributed and added as a constant.

This approach significantly reduces the number of photon track histories that need to be simulated in the Monte Carlo part, since the dose can be scored on a macroscopic grid. Additionally, the restriction to the simulation of photons avoids computational intensive simulations of electron trajectories on the micrometre scale. The microscopic distribution of doses is calculated voxel by voxel in a convolution approach and takes into account electron scattering only. As compared to pure convolution algorithms, there are no (known) material inhomogeneities within a voxel and therefore inaccuracies close to material interfaces are avoided. Figure 2 illustrates the generation of a microbeam dose profile with analytic electron dose kernels.

### 2.2. Monte Carlo approach for photon scattering

In the first stage of the algorithm, the transport of photons through the target geometry is modelled as a Monte Carlo simulation. The geometry for the simulation is based on a voxelized CT representation of the patient, with the conversion from Hounsfield units to tissue composition performed according to Schneider *et al* (2000).

Since the aim of this publication is a comparison of the new algorithm to an established Monte Carlo technique, a simple model of a radiation source has been chosen for better comparability of both techniques. The radiation source model can be refined for future adaptations to specific radiation sources. Investigations on the detailed simulation of a synchrotron radiation source can be found elsewhere (Martínez-Rovira *et al* 2012, Cornelius *et al* 2014).

The radiation source is described as a plane that emits photons perpendicular to its surface with homogeneous intensity. The shape of the radiation field may for example be defined by an absorber mask that outlines the microbeam field as a polygon. The radiation field outline for conformal irradiations is converted from a list of 2D points defining the polygon to a pixel grid for the simulation. Photons are emitted from all pixels whose centre is located inside the radiation field outline. The photon energy is sampled from the corresponding energy spectrum of the source. With MRT being currently exploited almost exclusively at synchrotron sources, the source to target distance is large (∼40 m) (Martínez-Rovira *et al* 2012) and thus the beam divergence at the target position is low. Therefore, it is justified to ignore the beam divergence in the simulation (Bartzsch *et al* 2014) and assume parallel beams. The photon polarization has a negligible effect on in-field doses in MRT (Bartzsch *et al* 2014) and is therefore not taken into account in the simulation of photon transport.

Interactions of photons with a kinetic energy of around 100 keV, are limited to the photoelectric effect, Compton scattering and Rayleigh scattering. Pair production processes are kinetically impossible in the orthovoltage energy range. Secondary electrons arising from photon interactions are not tracked in the simulation. The emission of bremsstrahlung photons of relevant kinetic energies by interactions of low-energy secondary electrons with light atoms of biological material is very infrequent and therefore the neglect of bremsstrahlung photons is assumed to have no significant impact on the simulation.

Since the maximum expected range of secondary electrons is of the order of hundreds of *μ*m and thus low compared to the voxel size of typically 1 mm–2 mm, all energy transfer from photons is assumed to be locally absorbed at the interaction point. The scored energy deposition is separated into two datasets for energy loss from primary interactions, i.e. the first interaction of a photon (primary dose) and secondary interactions, i.e. all subsequent interactions (scatter dose), and is divided by the local mass density and multiplied by the voxel volume to obtain the dose. This separation is necessary, since only the primary dose contributes to the microbeam pattern and the peak dose. The photon scatter dose leads to a valley dose contribution, which is approximately constant within the spatial scale of a CT voxel. The further processing of these two data sets is described in section 2.3.

The Monte Carlo photon transport is implemented in Geant4 (version 10.01.p01). Comparisons of full Monte Carlo simulations of microbeam irradiations have shown the equivalence of the Penelope and the Livermore physics models of Geant4, while the low energy standard physics model underestimates the dose deposition in the valley region (data not shown), which is in accordance with previous investigations comparing low energy standard physics and the Penelope physics model (Spiga *et al* 2007). Due to its better time efficiency the Penelope physics model was chosen for the photon transport. All secondary electron tracks are killed upon generation.

### 2.3. Kernel based algorithms for electrons created in primary photon interactions

In the Monte Carlo calculation the energy transferred from photons to secondary electrons is scored in each voxel. In the relevant range of photon energies, there are two processes where photons transfer energy to electrons: photoelectric absorption and Compton scattering. We refer to these two processes as energy transfer events (ETEs).

The dose distribution on the micrometre scale needs to take the electron energy transport into account. The electron energy absorption is calculated for each voxel individually, applying a few reasonable assumptions: within a single voxel it is assumed that the voxel material is homogeneous, the photon spectrum and the beam intensity do not change when the beam passes through the voxel. Furthermore the beam divergence may not lead to any significant changes in the microbeam pattern.

Following previous definitions, dose kernels are defined as the spatial distribution of the fractional mean energy ${\rm d} E$ absorbed per mass element ${\rm d} m$ caused by a primary particle interaction at the origin (Ahnesjö *et al* 1987, Bartzsch and Oelfke 2013). We refer to the electron kernel $K_{{\rm el}}^{{\rm 3D}}(\boldsymbol{r})$ as the dose kernel of scattering electrons created in a primary photon interaction. As spectrum and material do not change, the electron kernel is also constant within the voxel.

The Monte Carlo primary dose scores ETEs of unscattered photons and hence these events occur on the initial photon beam path. Therefore, within a single voxel, ETEs of the primary dose are equally spread along the beam direction and perpendicular to the beam direction they are distributed according to the fluence profile created by the microbeam collimator. ETEs of the Monte Carlo scatter dose are equally spread throughout the voxel, since photon scattering occurs on much larger spatial scales. Hence the scatter dose leads to a homogeneous dose bath and only electrons in primary interactions contribute to the microbeam pattern. Under these conditions the dose distribution in a single voxel can be calculated via (Debus *et al* 2017)

1 \begin{align*} \newcommand{\e}{{\rm e}} \displaystyle D(\boldsymbol{r})=D_{{\rm Scatter}}(\boldsymbol{r})+D_{{\rm Primary}}(\boldsymbol{r})\cdot(K_{{\rm el}}^{{\rm 3D}}(\boldsymbol{r}) * \nu(\boldsymbol{r})), \nonumber \end{align*}

where $\nu(\boldsymbol{r})$ describes the distribution of ETEs in the voxel, $D_{{\rm Scatter}}$ and $D_{{\rm Primary}}$ are the Monte Carlo primary and scatter dose contributions and $*$ denotes the convolution operator.

Within a CT-voxel the distribution of ETEs will not change along the beam propagation direction, as there is no beam divergence and absorption does not change the beam intensity within the voxel. Hence, by choosing the coordinate system in the voxel such that the propagation direction of the microbeams points along the *z*-axis the distribution function *ν* becomes independent of *z*. For planar microbeams in the *x*–*z*-plane *ν* depends on *y* only. Therefore the convolution can be significantly simplified and becomes either

2 \begin{align*} \newcommand{\e}{{\rm e}} \displaystyle D(x, y)=D_{{\rm Scatter}}(x, y)+D_{{\rm Primary}}(x, y)\cdot(K_{{\rm el}}^{{\rm 2D}}(x, y) * \nu(x, y)), \nonumber \end{align*}

*ν* depends on *x* and *y* or

3 \begin{align*} \newcommand{\e}{{\rm e}} \displaystyle D(y)=D_{{\rm Scatter}}(y)+D_{{\rm Primary}}(y)\cdot(K_{{\rm el}}^{{\rm 1D}}(y) * \nu(y)), \nonumber \end{align*}

if *ν* depends on *y* only. The convolution kernels $K_{el}^{1D}$ and $K_{{\rm el}}^{{\rm 2D}}$ can be obtained from the 3D scattering kernel $K_{{\rm el}}^{{\rm 3D}}$ by integration,

4 \begin{align*} \newcommand{\e}{{\rm e}} \displaystyle \begin{array}{@{}rcl@{}} K_{{\rm el}}^{{\rm 2D}}(x, y) &amp;=&amp; \int_{-\infty}^{\infty}K_{{\rm el}}^{{\rm 3D}}(x, y, z)\;{\rm d} z{\rm{~and}} \nonumber \\ K_{{\rm el}}^{1D}(y) &amp;=&amp; \int_{-\infty}^{\infty}\int_{-\infty}^{\infty}K_{{\rm el}}^{{\rm 3D}}(x, y, z)\;{\rm d} x\;{\rm d} z, \end{array} \label{eqn:Kernel1D2D} \nonumber \end{align*}

respectively.

For electron energies between 10 keV and a few 100 keV electron scatter kernels can be derived from the Bethe–Bloch stopping power equation. A detailed derivation has been published previously (Debus *et al* 2017). The starting point is the approximation of the stopping power *S* of an electron with kinetic energy *E*

5 \begin{align*} \newcommand{\e}{{\rm e}} \displaystyle S = K\cdot E^{-\alpha/(1-\alpha)}, \nonumber \end{align*}

where *K* and *α* are constants. Fitting experimental data from Berger *et al* (2005) leads to $\alpha\approx0.415$, independent of the material. Under a few assumptions, such as isotropical electron scattering and homogeneous material (Debus *et al* 2017), the three dimensional electron kernel can be derived,

6 \begin{align*} \newcommand{\e}{{\rm e}} \displaystyle K_{{\rm el}}^{{\rm 3D}}(r) = \frac{E_0(1-\alpha)}{4\pi\sigma\rho r^2}\left(1-\frac{r}{\sigma}\right)^{-\alpha}{\rm{,~where~}} r \in [0, \sigma]. \label{eqn:Kernel3D} \nonumber \end{align*}

*σ* denotes the electron continuous slowing down approximation range, *r* is the distance from the ETE, *ρ* is the mass density of the material and *E*_0_ is the initial electron energy. The scatter kernel (6) is normalized to dose per single electron. Evaluating equation (4) leads to the two dimensional scatter kernel

7 \begin{align*} \newcommand{\e}{{\rm e}} \displaystyle K_{{\rm el}}^{{\rm 2D}}(s) = \frac{E_0(1-\alpha)}{2\pi\rho\sigma s}\int_0^{\cos^{-1}\left(\frac{s}{\sigma}\right)}\left(1-\frac{s}{\sigma\cos\phi}\right)^{-\alpha}{\rm d}\phi, \nonumber \end{align*}

where *s* stands for $s=\sqrt{x^2+y^2}$ and defining $I_{{\rm 2D}}(\,p)$ as

8 \begin{align*} \newcommand{\e}{{\rm e}} \displaystyle I_{{\rm 2D}}^\alpha(\,p) = \int_0^{\cos^{-1}(\,p)}\left(1-\frac{p}{\cos\phi}\right)^{-\alpha} {\rm d}\phi \nonumber \end{align*}

leads to the simple representation

9 \begin{align*} \newcommand{\e}{{\rm e}} \displaystyle K_{{\rm el}}^{{\rm 2D}}(s) = \frac{E_0(1-\alpha)}{2\pi\rho\sigma s}I_{{\rm 2D}}^{\alpha}\left(\frac{s}{\sigma}\right) \nonumber \end{align*}

of the two dimensional scatter kernel. Similarly the 1D kernel can be calculated as

10 \begin{align*} \newcommand{\e}{{\rm e}} \displaystyle K_{{\rm el}}^{{\rm 1D}}(y) = \frac{E_0(1-\alpha)}{2\rho\sigma}\int_0^{1-\frac{y}{\sigma}}\frac{x^{-\alpha}}{1-x}{\rm d} x, \nonumber \end{align*}

which can be written in the simple representation

11 \begin{align*} \newcommand{\e}{{\rm e}} \displaystyle K_{{\rm el}}^{{\rm 1D}}(y) = \frac{E_0(1-\alpha)}{2\rho\sigma}I_{{\rm 1D}}^\alpha\left(1-\frac{y}{\sigma}\right), \nonumber \end{align*}

identifying $I_{{\rm 1D}}^\alpha$ with

12 \begin{align*} \newcommand{\e}{{\rm e}} \displaystyle I_{{\rm 1D}}^\alpha(\,p) = \int_0^{\,p}\frac{x^{-\alpha}}{1-x}{\rm d} x. \nonumber \end{align*}

These derived kernels explicitly depend on the initial electron energy *E*_0_ and on the electron range *σ*, which is material and energy dependent. Electrons produced in photoelectric absorption are assumed to receive all of the primary photon energy $E_{{\rm ph}}$, neglecting the binding energy, while only a fraction *p* of the photon energy is transferred to electrons in Compton scattering,

13 \begin{align*} \newcommand{\e}{{\rm e}} \displaystyle E_0=pE_{{\rm ph}}. \nonumber \end{align*}

The ratio of photoelectric absorption to Compton scattering interactions defined by the ratio of their scattering coefficients depends on photon energy and material; *p* depends on photon energy, only. The electron kernel of a polychromatic photon beam with the power contributions *f*(*E*_*i*_) at photon energy *E*_*i*_ can be calculated as a weighted sum

14 \begin{align*} \newcommand{\e}{{\rm e}} \displaystyle K_{{\rm el}}(\boldsymbol{r}) = \sum\limits_i f(E_i)\frac{\mu_{\rm c}(E_i)K_{{\rm el}}(\boldsymbol{r}, E_0 = pE_i) + \mu_{\rm p}(E_i)K_{\rm el}(\boldsymbol{r}, E_0 = E_i)}{p\mu_{\rm c}(E_i)+\mu_{\rm p}(E_i)}, \nonumber \end{align*}

where $\mu_{\rm c}(E_i)$ and $\mu_{\rm p}(E_i)$ are the energy and material dependent scattering coefficients for Compton and photoelectric effect. This formula is valid for one, two and three dimensional scattering kernels.

### 2.4. Comparisons between Monte Carlo and hybrid algorithm

We compare the performance of the hybrid algorithm for various beam and sample geometries to pure Monte Carlo simulations in Geant4. Geant4 simulations of MRT have been compared to Penelope and radiochromic film measurements by Cornelius *et al* (2014), showing good agreement. Geant4 is therefore assumed to be representative of a state-of-the-art technique for MRT dose calculation.

First, planar microbeams with 50 *μ*m width and 400 *μ*m peak to peak spacing are projected onto a homogeneous cubic water phantom of 160 mm side length. The field size is $20 \times 20$ mm. To demonstrate the performance at various photon beam energies, mono-energetic beams are simulated: 50 keV, 100 keV and 200 keV, covering the relevant photon energies of the spectrum at the biomedical beamline ID17 of the ESRF in Grenoble (Crosbie *et al* 2015). Apart from that the polychromatic spectrum of ID17 is used for all of the following simulations.

In a second example pencil beams are simulated in the same cubic water phantom. A $20 \times 20$ mm field of a grid of 50 *μ*m side length squared pencil beams with a pitch of 400 *μ*m is simulated.

In the third example a cross firing at right angle of 50 *μ*m wide and 400 *μ*m spaced planar microbeams is simulated in the centre of the water cube. Again the field sizes are $20 \times 20$ mm.

The same fields are used to calculate the dose in a simplified head phantom. The phantom is cube shaped with 160 mm side length. A 4 mm thick layer of water below the surface of the cube models the skin, underneath is a 6 mm thick layer of bone. Otherwise the phantom is made of water except for a small cubic piece of bone with a side length of 10 mm. This piece of bone is positioned such that one corner is at the centre of the phantom. It is introduced to demonstrate the performance of the algorithm, when an inhomogeneity is present in the cross firing region of the microbeam fields.

Finally the performance of the hybrid algorithm is tested in a realistic anthropomorphic head phantom (Radiosurgery head phantom, CIRS, Norfolk, USA). The phantom imitates radiological properties and the shape of a human head. Material compositions are calculated based on Hounsfield units (HU) of an acquired CT of the phantom. Dose calculation is performed with three different algorithms for a $20 \times 20$ mm sized microbeam field with 50 *μ*m wide and 400 *μ*m spaced beams: pure Monte Carlo dose calculation, pure convolution based dose calculation and the hybrid algorithm. A comparison is performed to compare the accuracy of the hybrid approach with pure convolution algorithms.

Dose calculations with pure Monte Carlo methods are performed in Geant4 version 10.01.p01 with the Penelope physics model. A correct dose scoring on the micrometre scale is ensured by reducing the production threshold for secondary particle generation to 1 *μ*m and by forcing the propagation of electrons to a maximum step size of 5 *μ*m. The scoring resolution is 5 *μ*m lateral to the microbeams and 500 *μ*m along the microbeams and the propagation vector. For the pencil beam field 5 *μ*m scoring resolution was required in both dimensions perpendicular to the beam propagation vector, with secondary particle production thresholds of 1 *μ*m and a maximum electron step size of 5 *μ*m. The material composition and densities were chosen to be identical to those in the hybrid dose calculation.

The computation times for the Monte Carlo simulation stated in the results section are given for an Intel Xeon E5-2608v4 CPU (14 cores) with 28 parallel threads, unless stated differently. The hybrid algorithm dose calculations were performed on two Intel Xeon E5-2690v4 CPUs (14 cores) at 2.6 GHz with 56 parallel threads.

## 3. Results

### 3.1. Microbeams in homogeneous water

**Figure 3.**
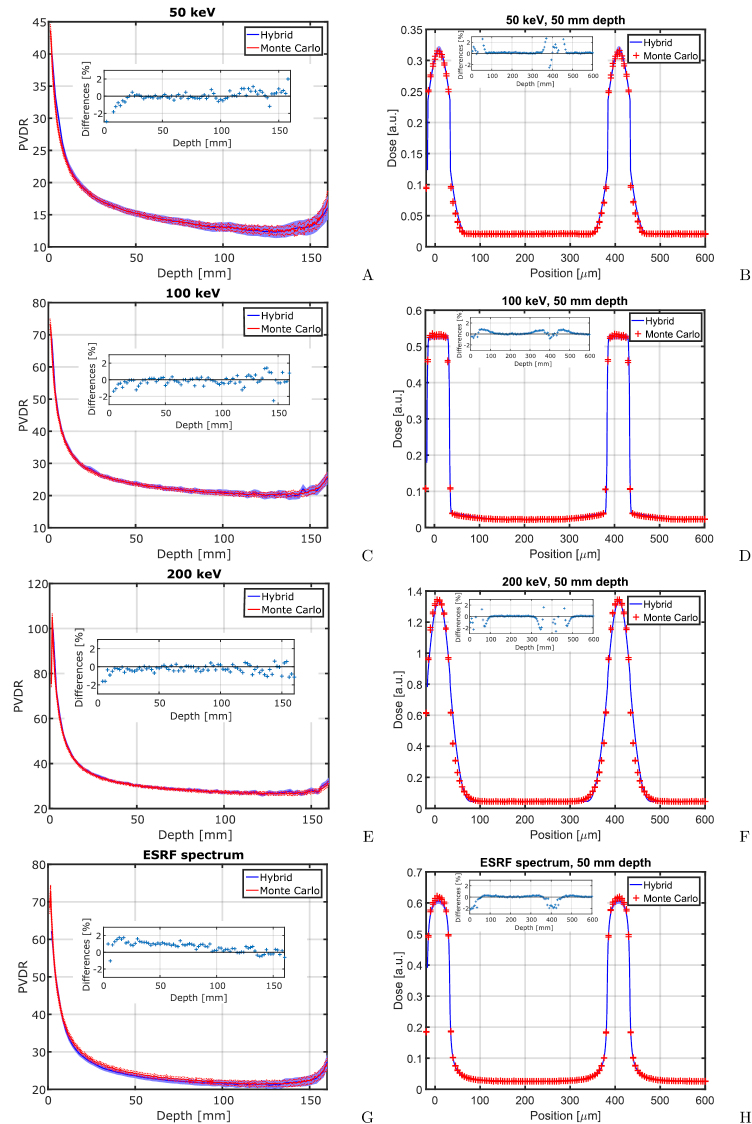
The figure shows the PVDR over depth (left column) and the profiles of planar microbeams at 50 mm depth. Calculations with the hybrid algorithm (solid line) are compared to pure Monte Carlo calculations (‘+’) for 50 keV, 100 keV and 200 keV microbeams, and microbeams with the ESRF spectrum in the first, second, third and fourth rows, respectively. Small inserts show relative differences between the Monte Carlo and hybrid algorithm. They are positive if the Monte Carlo results are larger than values calculated with the hybrid algorithm. 95% confidence intervals are provided for all calculations that involve Monte Carlo simulations.

In figure 3 calculated PVDRs and beam profiles of planar 50 *μ*m wide and 400 *μ*m spaced microbeams are compared between hybrid algorithm and pure Monte Carlo simulations. The differences between the Monte Carlo and hybrid algorithm calculated PVDRs are less than 5%. The Monte Carlo calculated shapes of the microbeam profiles agree closely with the predictions of the hybrid algorithm. Figure 3 illustrates changes in the physics of dose absorption with increasing energy. At 50 keV kinetic energy, photons frequently interact via photoelectric absorption, which accounts for around 13% (Berger *et al* 2010) of all photon interactions. Secondary electrons originating from photoelectric absorption have an initial kinetic energy of 50 keV and consequently their range of 43 *μ*m (Berger *et al* 2005) is comparably large. Electrons created in Compton scattering have kinetic energies of typically less than 10 keV and are absorbed within less than 5 *μ*m from their creation. Due to scattering, energy transferred to electrons in the microbeam peaks is transported into the valley and smears out the microbeam edges. At 100 keV the fraction of photons interacting via photoelectric absorption has significantly decreased to less than 2%. Secondary electrons are mainly created in Compton scattering events, but receive on average only 13 keV energy from the scattering photon. These electrons have a range of just around 4 *μ*m. Therefore the beam penumbras are steep and only a small fraction of photoelectrons scatters further. At 200 keV beam energy the photoelectric absorption is negligible and photons interact almost exclusively by Compton scattering, transferring on average 44 keV kinetic energy to secondary electrons, which have a range of more than 30 *μ*m. In contrast to the 50 keV beam there are almost no short ranged electrons and therefore the steep parts of the profile disappear.

For all simulated energies the relative difference between Monte Carlo simulations and hybrid algorithm are below 2%, except for the beam entrance of the 50 keV beam, where the difference is at around 3%. Higher differences in the beam entrance region are expected due to steep dose gradients, especially at low photon energies. For the ESRF spectrum the hybrid algorithm calculates on average between 1 and 2% lower peak doses and hence PVDRs than Monte Carlo simulations. For all profiles relative differences of peak and valley doses between the Monte Carlo simulation and the hybrid are below 2%. However, there are larger deviations at the beam penumbras. In particular at 200 keV photon energy, the hybrid algorithm is less accurate at the far edge of the beam profile due to simplifications with respect to the spectrum of the Compton electrons and range straggling, such that between 50 *μ*m and 65 *μ*m the distance from the beam centre the dose is underestimated by around 2% in the hybrid dose calculation as compared to Monte Carlo.

For the ESRF spectrum, figures 3(G) and (H) show the PVDR depending on depth in water and the microbeam profiles at 50 mm depth, respectively. Peak doses calculated with the hybrid dose calculation are up to 3.5% lower than those calculated with a pure Monte Carlo approach. These differences are caused by high energy contributions of the synchrotron spectrum, where the approximations made in the derivation of electron scatter kernels are less accurate.

The calculation times for these data sets are 76.0 h (50 keV, $4.9 \cdot 10^{10}$ photon histories), 99.3 h (100 keV, $4.8 \cdot 10^{10}$ photon histories), 251.4 h (200 keV, $4.9 \cdot 10^{10}$ photon histories), and 112.5 h (ESRF spectrum, $4.7 \cdot 10^{10}$ photon histories) for the Monte Carlo simulation and 14 m (50 keV), 14 m (100 keV), 15 m (200 keV), and 19 m (ESRF spectrum) for the hybrid algorithm with $1 \cdot 10^{8}$ photon histories each.

### 3.2. Pencil beams

**Figure 4.**
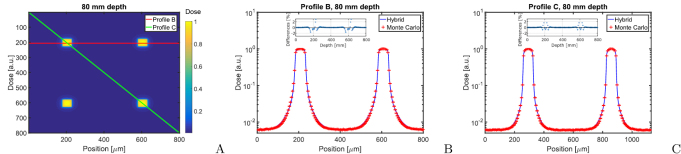
The figure shows a comparison of pencil beam profiles in 80 mm depth in a homogeneous water phantom. (A) presents a dose heat map in 80 mm depth and indicates the position of the horizontal and diagonal profiles shown in (B) and (C). Small inserts show the relative differences between the Monte Carlo and hybrid algorithm.

The result of the pencil beam simulations are shown in figure 4. For the calculation of the pencil beam profiles the two dimensional electron scatter kernels are used. Differences between Monte Carlo and hybrid algorithm are less than 1% in the peak and valley. Differences in the beam penumbra fall-off can reach up to 8% in dose or 5 *μ*m in position and peak at around 80 *μ*m distance from the beam centre.

Calculation times are 71.3 h (Monte Carlo, $1.74 \cdot 10^{11}$ photon histories) and 19 m (hybrid, $1 \cdot 10^{8}$ photon histories).

### 3.3. Cross firing geometries

**Figure 5.**
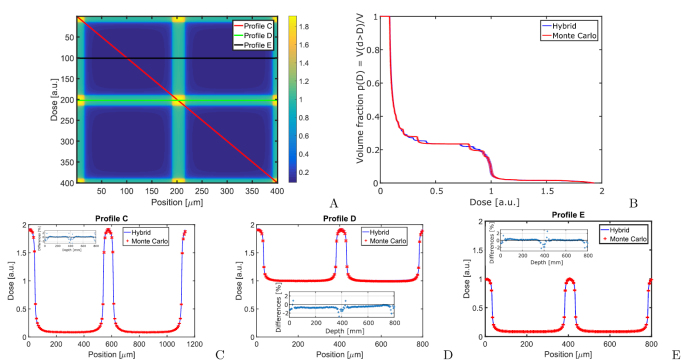
The figure shows the dose distribution in the cross firing region of two microbeam fields with planar microbeams of 50 *μ*m width and 400 *μ*m spacing. A shows a colour scale overview, while (B) presents the DVH in the displayed cross firing region. Figures (C)–(E) show the dose profiles according to (A). Small inserts show the relative differences between the Monte Carlo and hybrid algorithm.

Figure 5 presents Monte Carlo and hybrid algorithm calculated doses in the cross firing region of two microbeam fields in the centre of a cubic water phantom. The data is also presented in cumulative dose–volume histograms (DVH) in figure 5(B). As in conventional radiotherapy the DVH presents on the vertical axis the fraction of the volume that receives at least the dose on the horizontal axis. For complicated beam geometries the DVH presents a useful way to visualize volume fractions that receive certain dose levels. Except for partial volume effects the hybrid algorithm and Monte Carlo calculations produce equivalent DVHs in the cross firing region. Dose differences between the Monte Carlo and hybrid algorithm are below 4% in all profiles. The highest uncertainties arise in the beam penumbra regions and can be attributed to partial volume effects. Within the peak and valley plateau region differences are below 2%.

The calculation times are 164.3 h (Monte Carlo, $4.16 \cdot 10^{11}$ photon histories) and 30 m (hybrid, $2 \cdot 10^{8}$ photon histories).

### 3.4. Cross firing geometry in an inhomogeneous phantom

**Figure 6.**
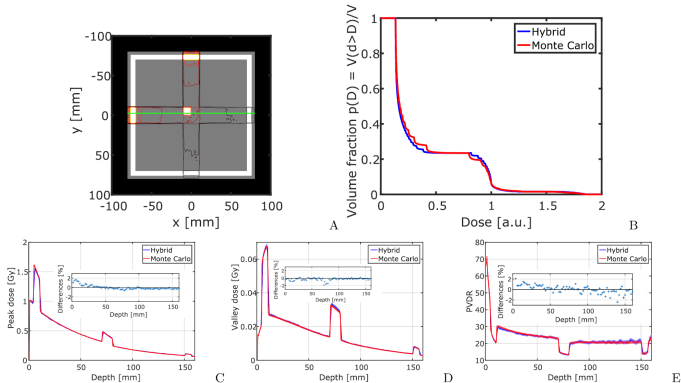
Cross firing of two microbeam fields in an inhomogeneous phantom. (A) shows how the two fields intersect in the centre of the phantom. (B) is the DVH of the cross firing region within the bone. (C)–(E) show peak dose, valley dose and PVDR dependence on the penetration depth in the phantom for one of the two fields as indicated by the line in figure (A). Small inserts show relative differences between the Monte Carlo and hybrid algorithm.

Figure 6 illustrates Monte Carlo and hybrid algorithm generated dose calculation results in an inhomogeneous phantom. Two microbeam fields were irradiated perpendicular to each other and meet in the centre of the phantom, where a small volume of bone is situated. The DVH in figure 6(B) records the doses distribution within the this piece of bone. Partial volume effects lead again to step shaped DVH curves, in particular for the Monte Carlo calculated dose distributions, where voxel sizes are larger (5 *μ*m pitch). Apart from the partial volume effects, the peak and valley doses agree within 2%. Figures 6(C) and (D) show peak and valley doses, and PVDRs for one of the microbeam fields, as indicated by the profile line in figure 6(A). Differences between hybrid algorithm and Monte Carlo are below 2% in the peak and below 2.5% in the valley. Differences in the PVDR are less than 2%. The hybrid algorithm does not lead to over- or underestimation of valley doses close to material interfaces and therefore substantially outperforms purely convolution based dose calculation approaches in accuracy.

The calculation times are 285.0 h (Monte Carlo, one beam, $1.18 \cdot 10^{11}$ photon histories) and 29 m (hybrid, 2 beams, $2\cdot 10^8$ photon histories).

### 3.5. Microbeams in an anthropomorphic head phantom

**Figure 7.**
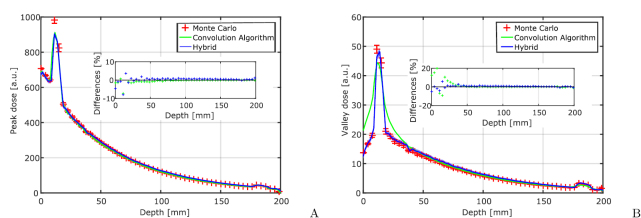
The figure shows peak (A) and valley (B) doses for a $20 \times 20$ mm microbeam field that was irradiated into an anthropomorphic head phantom. Doses were calculated with Monte Carlo simulations, the convolution algorithm and the hybrid algorithm. Error bars for the Monte Carlo simulations indicate 95% confidence intervals. Small inserts show relative differences between the Monte Carlo and hybrid algorithm.

The improvement of the hybrid approach compared to a purely kernel based approach is shown when comparing hybrid dose calculation, the Monte Carlo and the pure convolution algorithm results in dose calculations of microbeams in an anthropomorphic head phantom. The calculation results are presented in figure 7. There is a general good agreement of peak doses between the three dose calculation methods, with the exception of the Monte Carlo calculated peak dose in the skull close to the beam entrance region. Otherwise peak dose differences remain below 3%. For the valley doses there is a good agreement between the Monte Carlo and hybrid dose calculation with differences below 5%. The largest deviations appear in the region of strong dose gradients close to the skull. The purely convolution based dose calculation approach shows large differences in the valley dose of up to 20% close to the skull and beam entrance region. The valley dose in the skull is underestimated by 8%–10% using the convolution algorithm. In the homogeneous parts of the phantom, valley doses calculated with the convolution algorithm are in agreement with the Monte Carlo and hybrid algorithm.

The calculation times are 107 h (Monte Carlo), 3.7 m (convolution algorithm from Debus *et al* (2017), calculations on an Intel Core i7-4770 CPU with 3.4 GHz), and 20 m (hybrid, $2 \cdot 10^8$ photon histories).

## 4. Discussion

Calculation times of the developed hybrid dose calculation algorithm are significantly lower than for pure Monte Carlo simulations. It is difficult to quote computation times, since they depend on the required accuracy, resolution, field size and also computer architecture. With the hybrid algorithm dose calculations for human sized phantoms on a CT grid with 2 mm resolution, inaccuracy of less than 3% and a single microbeam field of $20 \times 20$ mm size as used in this manuscript can typically be performed within half an hour, while pure Monte Carlo computations require calculation times of the order of days. The hybrid algorithm is slower than a purely kernel based dose calculation (Debus *et al* 2017), which usually takes around 5 min. However, the gain in accuracy at material interfaces justifies these moderately increased calculation times with the hybrid dose calculation method.

There are several possibilities to speed up the dose calculation. The Monte Carlo part of the hybrid method is still the slowest part of the calculation. However, Monte Carlo simulations are based on the Geant4 Monte Carlo toolkit. Faster Monte Carlo algorithms are available such as those presented by Jia (2012), and may actually compete in speed with pure kernel based algorithms.

The results presented in this manuscript demonstrate that pure Monte Carlo and hybrid calculated dose distributions show satisfying equivalence. Observed differences concern mainly minor changes in the microbeam penumbras, which are unlikely to impact on therapy outcome.

Far more important is the material composition deduced from the CT image. Especially at low photon energies, small differences in the fraction of high Z atoms can lead to substantial changes in the absorption and scattering of photons. This uncertainty is, however, independent of the dose calculation method.

Another limitation of the described method is that small anatomical structures are not taken into account if they are smaller than the voxel size. Recently the application of microbeams for lung tumours has been discussed (Wright 2015). The alveoli in the lung are air filled cavities are approximately 100 *μ*m in size and can deteriorate microbeam dose distributions in the organ. The simple assumption of homogeneous mix of air and water may not be sufficient to describe the dose distribution in lung tissue. However, it should be noted that currently neither Monte Carlo nor any other dose calculation method is capable of calculating this effect, since the necessary structural information on the micrometre scale is not available.

## 5. Conclusion

In this manuscript we introduced a hybrid dose calculation method for microbeam radiation therapy that combines Monte Carlo calculation of photon scattering and kernel based calculation of electron scattering. It was demonstrated that this approach is an efficient and accurate way to calculate even complicated cross-firing geometries of spatially modulated radiation fields.

A reliable and fast dose calculation tool is a prerequisite for clinical trials in microbeam radiation therapy. The new algorithm has shown to be as accurate as a full Monte Carlo simulation, while being substantially more efficient in the use of computing resources in all presented cases.

Future extensions of the developed algorithm may include more complicated source models and polarization effects (Bartzsch *et al* 2014). Also divergent radiation fields can in principle be computed with the aid of the hybrid algorithm.
